# Infrared spectroscopy with multivariate analysis to interrogate endometrial tissue: a novel and objective diagnostic approach

**DOI:** 10.1038/sj.bjc.6606094

**Published:** 2011-02-15

**Authors:** S E Taylor, K T Cheung, I I Patel, J Trevisan, H F Stringfellow, K M Ashton, N J Wood, P J Keating, P L Martin-Hirsch, F L Martin

**Affiliations:** 1 Centre for Biophotonics, Lancaster Environment Centre, Lancaster University, Bailrigg, Lancaster LA1 4YQ, UK; 2 Lancashire Teaching Hospitals NHS Trust, Royal Preston Hospital, Sharoe Green Lane North, Fulwood, Preston, Lancashire PR2 9HT, UK

**Keywords:** attenuated total reflection Fourier-transform infrared spectroscopy, endometrial cancer, endometrium, infrared spectroscopy, multivariate analysis

## Abstract

**Background:**

Endometrial cancer is the most common gynaecological malignancy in the United Kingdom. Diagnosis currently involves subjective expert interpretation of highly processed tissue, primarily using microscopy. Previous work has shown that infrared (IR) spectroscopy can be used to distinguish between benign and malignant cells in a variety of tissue types.

**Methods:**

Tissue was obtained from 76 patients undergoing hysterectomy, 36 had endometrial cancer. Slivers of endometrial tissue (tumour and tumour-adjacent tissue if present) were dissected and placed in fixative solution. Before analysis, tissues were thinly sliced, washed, mounted on low-E slides and desiccated; 10 IR spectra were obtained per slice by attenuated total reflection Fourier-transform IR (ATR-FTIR) spectroscopy. Derived data was subjected to principal component analysis followed by linear discriminant analysis. Post-spectroscopy analyses, tissue sections were haematoxylin and eosin-stained to provide histological verification.

**Results:**

Using this approach, it is possible to distinguish benign from malignant endometrial tissue, and various subtypes of both. Cluster vector plots of benign (verified post-spectroscopy to be free of identifiable pathology) *vs* malignant tissue indicate the importance of the lipid and secondary protein structure (Amide I and Amide II) regions of the spectrum.

**Conclusion:**

These findings point towards the possibility of a simple objective test for endometrial cancer using ATR-FTIR spectroscopy. This would facilitate earlier diagnosis and so reduce the morbidity and mortality associated with this disease.

Attenuated total reflection Fourier-transform infrared (ATR-FTIR) spectroscopy allows the objective classification of biological material on a molecular level. Previous studies have shown the potential of vibrational spectroscopy in categorising cancer and intraepithelial neoplasia in tissues such as the cervix, prostate, and various parts of the gastrointestinal tract (Sindhuphak *et al*, 2003; German *et al*, 2006; Maziak *et al*, 2007; Walsh *et al*, 2008; Baker *et al*, 2009; Sahu *et al*, 2010). Many of these tissues are classified and analysed as either normal or abnormal. In contrast, human endometrium is a tissue that exhibits biological plasticity, capable of adopting several different histological conformations depending on the hormonal milieu.

Endometrial cancer is the most common gynaecological malignancy in the United Kingdom. This is most likely to be related to the increasing prevalence of obesity among women (Renehan *et al*, 2008). Adipose tissue contains the enzyme aromatase, responsible for the conversion of the adrenal-derived androgen androstenedione to oestrone, an oestrogen. This acts unopposed by progesterone to stimulate endometrial proliferation in perimenopausal and postmenopausal women (Schindler, 2009). Oestrone also appears to exert genotoxic effects (Yared *et al*, 2002). Endometrial cancer is frequently underestimated as a fairly benign condition. Indeed, early, low-grade disease has an excellent prognosis; however, a significant minority of patients are less fortunate and have advanced or high-grade disease (Uharcek, 2008).

Endometrial cancers are classified according to their behaviour and histological appearance into two groups. Endometrioid cancers are often referred to as type I disease. They are associated with obesity, polycystic ovarian syndrome, unopposed oestrogen hormone replacement therapy, and the perimenopausal age group. All of these factors contribute to a relative oestrogen excess (Bokhman, 1983). Endometrioid cancers are graded 1–3 according to the proportion of solid tissue within the tumour, with grade 3 containing the least glandular tissue and being the most aggressive (Horn *et al*, 2007). Type II endometrial cancers include the uterine serous and clear cell subtypes. These are found in older postmenopausal women, are unconnected with oestrogen, and behave in a more aggressive manner. Carcinosarcomas are frequently included in this category; however, their true relation to other uterine cancer subtypes is uncertain (D'Angelo and Prat, 2010). Type I and II endometrial cancers have different mutation profiles and precursor lesions (Sherman, 2000; Fadare and Zheng, 2008).

Biomolecules absorb energy in the mid-infrared (IR) region of the electromagnetic spectrum (Martin *et al*, 2010). Attenuated total reflection Fourier-transform IR spectroscopy exploits this property, measuring the absorbance of IR within this region. Each individual chemical bond has its own vibrational characteristics, causing it to absorb IR at a particular frequency (usually expressed as wavenumber=1/wavelength). Plotting wavenumber against intensity of absorbance generates a spectrum that represents the quantity and type of bonds present within the material examined. When applied to biological material this distinctive pattern is known as the ‘biochemical-cell fingerprint’. Changes in the type, conformation, or quantity of bonds generate different spectra; this could be used to differentiate between benign and malignant tissues. Powerful computational data reduction and separation techniques are required to interpret the vast quantity of data generated (Martin *et al*, 2007). Here, principal component analysis (PCA) followed by linear discriminant analysis (LDA) has been used. Principal component analysis identifies variance and compresses the data. Linear discriminant analysis demonstrates separation between different categories such as histological types. Combined together, these two techniques reveal the chemical bonds that are most important in such separation.

In previous work, transmission FTIR microspectroscopy was applied using a synchrotron source to de-waxed paraffin-embedded endometrial tissue (Kelly *et al*, 2009). For endometrial spectroscopy to have a practical application, it must be simple and function without the need for a particle accelerator. Attenuated total reflection Fourier-transform IR spectroscopy fulfils these requirements. This is the first description of ATR-FTIR spectroscopy in endometrial tissue and addresses the potential applications of this technique in the future.

## Patients and Methods

### Study participants

Informed consent to take endometrial tissue for use in research was obtained from women undergoing hysterectomy. Ethical committee approval was obtained (LREC no. 05/Q1302/83; Preston, Chorley and South Ribble Ethical Committee). Patients were allocated study numbers based on their preoperative diagnostic category. In total, 101 endometrial samples were obtained from 76 patients, 36 of whom had endometrial cancer (see Table 1). The indications for hysterectomy in the benign group included bleeding problems (dysfunctional uterine bleeding, *n*=6), pelvic mass (fibroids, *n*=6; ovarian mass, *n*=7), pelvic pain (endometriosis, *n*=4; adenomyosis, *n*=6; endometriosis and adenomyosis, *n*=2), premalignant disease (cervical intraepithelial neoplasia, *n*=2; suspected hyperplasia, *n*=2), and prophylaxis (previous breast cancer, *n*=5).

### Tissue preparation

After surgical resection, unfixed hysterectomy specimens were transported directly to the pathology laboratory. Here, a consultant histopathologist dissected out a sliver of endometrial tissue. If the uterus contained tumour, a piece of this was sampled together with a piece of adjacent macroscopically benign endometrium (if present); otherwise, a representative piece of endometrium was chosen. Tissue samples were then placed in Surepath cytology medium. The time from removal of the uterus from the patient to placement of the sample in fixative was <20 min.

**Figure 1:**
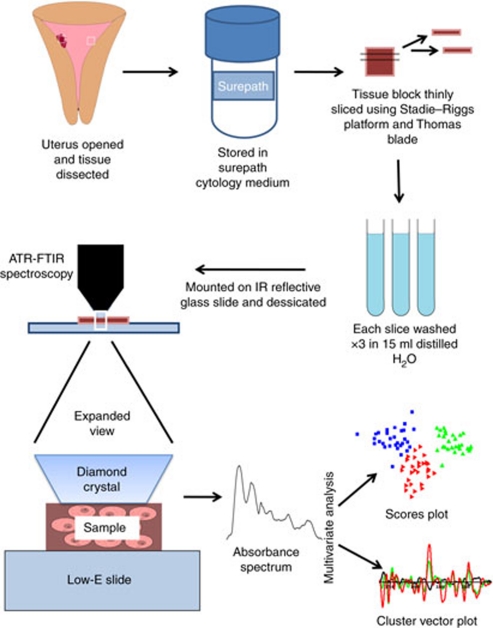
Diagrammatic representation of methodology.

All samples were stored in Surepath at room temperature for between 48 h and 4 weeks. After fixation, tissue samples were thinly sliced (⩽1-mm thick/slice) by hand using a Stadie–Riggs platform and Thomas blade (Martin and McLean, 1995). Two slices were prepared from each endometrial specimen. The tissue samples were orientated so that the first slice consisted of the surface of the endometrium and the second, the tissue immediately below. Each slice was washed in three separate 10-ml volumes of distilled H_2_O. Slices were mounted on low-E reflective glass slides and placed in a desiccator for a minimum of 48 h. An overview of the methodology is shown in Figure 1.

### Attenuated total reflection Fourier-transform infrared spectroscopy

Infrared spectra were obtained using a Bruker Vector 22 FTIR spectrometer with Helios ATR attachment containing a diamond crystal (Bruker Optics Ltd, Coventry, UK); 10 spectra were acquired per slide, each from a different area of the tissue slice. The ATR crystal was cleaned with dry tissue paper between each spectra acquisition, and a background reading was taken prior to each new slide. A cut-off value for the Amide I peak was designated as 0.12, to ensure spectral quality (Trevisan *et al*, 2010). Spectra were cut to between 1800 and 900 cm^−1^, baseline corrected and normalised to Amide I (1650 cm^−1^). Because of the non-destructive nature of this bio-analytical approach, the post-spectroscopy sections were then stained with haematoxylin and eosin (H&E). Macroscopically normal endometrium sometimes contains important lesions (intraepithelial carcinoma, subtle areas of hyperplasia, and carcinoma restricted to the endometrium) identifiable under the microscope. It is not possible to totally validate a tissue as normal or benign; however, this allowed histological categorisation and verification of the tissue sections analysed in this study.

### Computational analysis

Multivariate analysis was performed with PCA followed by LDA using MATLAB R2008a software (The Mathworks Inc., Natick, MA, USA). Principal component analysis was used for preliminary data reduction and to demonstrate variance in an unsupervised manner. Linear discriminant analysis derives vectors from the principal components (PCs) and so minimises the within-category differences (which would mostly be associated with typical heterogeneity in any tissue sample) while maximising between-category discriminating characteristics (i.e., those most likely to be diagnostic). This allows separation between groups to be visualised more clearly. Multivariate analysis results are visualised in two different ways: scores plots and cluster vector plots. Scores plots are scatter charts drawn using the data values obtained after PCA–LDA (i.e., the ‘scores’) as Cartesian coordinates. Cluster vector plots are ‘pseudo-spectra’ (in the sense that as spectra, they contain values per wavenumber, although they contain coefficients of linear combinations) obtained by the following construction: each cluster vector is associated with a different histology type, it corresponds exactly to the vector that points from the mean spectrum of a reference histology type to mean spectrum from its specific histology type (Martin *et al*, 2007; Llabjani *et al*, 2010). This construction is done inside the vector space spanned after the PCA–LDA data reduction. These techniques were applied to total spectra per slide. All wavenumbers are rounded down to the nearest whole number.

## Results

Principal component analysis–LDA of the biochemical-cell-fingerprint region (1800–900 cm^−1^) was used to derive scores plots and corresponding cluster vector plots for several combinations of tissue types. Principal component analysis–LDA scores plots display each spectrum as a point in multidimensional space (number of dimensions is determined by *n*−1, where *n* is the number of groups being compared) with proximity implying spectral, and therefore biochemical, similarity. The cluster vector plots also display variance, but here it is shown as the difference between one group (plotted as the baseline) as compared with all other groups at all wavenumbers.

### Tracking normal tissue through the menstrual cycle

**Figure 2:**
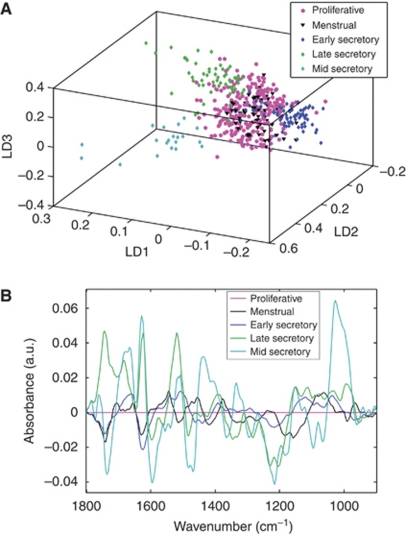
Comparison of normal premenopausal endometrial tissue subtypes through the menstrual cycle. (**A**) Rotated scores plot. (**B**) Cluster vector plot (proliferative tissue as comparator).

Normal endometrium, obtained from premenopausal women, was classified by the histological phase of the menstrual cycle. Clustering within and separation between most phases was observed. This was most clearly visible after rotation of the three-dimensional (3D) plot (Figure 2A). Clear separation is seen between the three subgroups of secretory endometrium. Proliferative phase endometrium and menstrual phase endometrium did not separate. The cluster vector plot shown in Figure 2B uses proliferative endometrium as the baseline and all others are compared with this. The phases adjacent to the proliferative phase in the cycle (menstrual and early secretory) are the most biochemically similar to it. Late secretory endometrium exhibits large peaks in the lipid (1743 cm^−1^), Amide I (1624 cm^−1^), Amide II (1519 cm^−1^), and asymmetric phosphate stretching vibrations (*ν*_as_PO_2_^−^ (1203 cm^−1^)) regions. Mid-secretory endometrium also has prominent peaks in the lipid region (1735 cm^−1^), Amide I (1666, 1627, 1597 cm^−1^), Amide II (1516 cm^−1^), other protein regions (1481, 1435 cm^−1^), *ν*_as_PO_2_^−^ (1215 cm^−1^), and symmetric phosphate stretching vibrations (*ν*_s_PO_2_^−^ (1095 cm^−1^)) regions. The largest peak is at 1026 cm^−1^; this corresponds with glycogen, a biochemical substrate known to be present at high levels in mid-secretory endometrium (Robles *et al*, 1972).

### Benign tissue *vs* cancer

**Figure 3:**
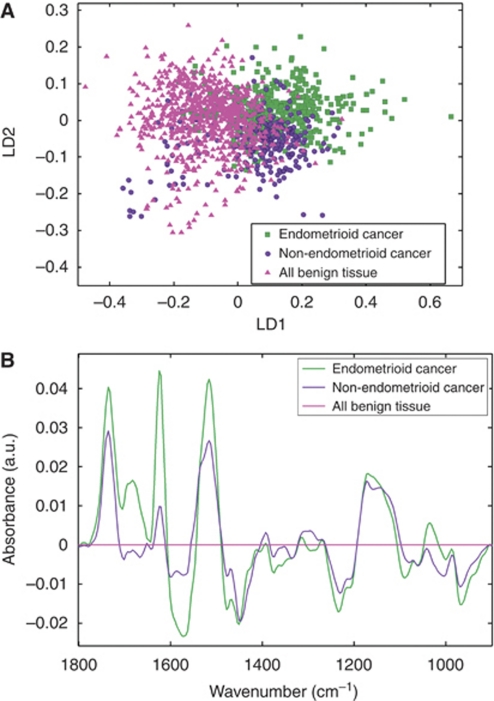
Comparison of malignant subtypes (endometrioid and non-endometrioid) with benign endometrial tissue. (**A**) Scores plot. (**B**) Cluster vector plot (benign tissue as comparator).

Benign and malignant endometrial tissues were compared. The benign group consisted of tissues from all the women recruited to the study who did not have endometrial cancer. After spectroscopy analyses, these tissue sections were H&E-stained; in all cases, the designated tumour sections contained cytological atypia and altered tissue architectural characteristics, whereas the designated benign sections appeared normal, although postmenopausal tissues typically showed an absence of glandular elements. The tumour tissues were subdivided into endometrioid cancer and non-endometrioid cancer. Figure 3A shows the scores plot for these three categories. There is a degree of overlap between them, but ∼80% of separation between benign and malignant spectra can be achieved by drawing a line perpendicular to LD1, near the point of origin. Figure 3B shows the cluster vectors of the two malignant classes *vs* benign tissue. The majority of the difference between benign and malignant tissue lies in the lipid (1735 cm^−1^) and Amide I/II (1624, 1570, 1516 cm^−1^) regions of the spectra, although there are also important contributions from a protein band (1477 cm^−1^), *ν*_as_PO_2_^−^ (1230 cm^−1^), RNA/carbohydrate (1168 cm^−1^), and phosphorylated proteins (968 cm^−1^).

Endometrioid cancers and non-endometrioid cancers are spectrally similar between 1500 and 900 cm^−1^ as the cluster vector plots exhibit similar patterns, although several peaks are shifted by a few wavenumbers. Both have peaks at around 1450 cm^−1^, with further peaks at 1230 cm^−1^ (*ν*_as_PO_2_^−^) and 1168 cm^−1^ (RNA/carbohydrate). Between 1800 and 1500 cm^−1^, the vectors differ in magnitude of absorbance, with larger lipid (1735 cm^−1^), Amide I (1624 cm^−1^) and Amide II (1570, 1516 cm^−1^) peaks together with a separate peak at 1681 cm^−1^ (Amide I) for endometrioid cancers (see Table 2 for a full list of distinguishing wavenumbers).

### Benign tissue *vs* tumour and tumour-adjacent tissue

**Figure 4:**
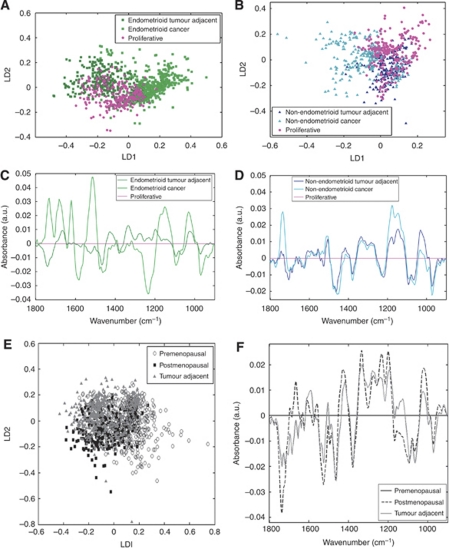
Comparison of cancer tissue with corresponding tumour-adjacent tissue and normal proliferative endometrium. (**A**) Endometrioid scores plot. (**B**) Non-endometrioid scores plot. (**C**) Endometrioid cluster vector plot (proliferative as comparator). (**D**) Non-endometrioid cluster vector plot (proliferative as comparator). (**E**) All benign tissue scores plot. (**F**) All benign tissue cluster vector plot (premenopausal as comparator).

Proliferative endometrial tissue was compared with tumour tissue and its corresponding tumour-adjacent macroscopically normal tissue (verified as such following microscopic examination of post-spectroscopy H&E-stained sections). Figure 4A and C show endometrioid cancer compared with corresponding tumour-adjacent tissue compared with proliferative tissue for cluster segregation in the scores plot and discriminating features in the loadings plot. Likewise, Figure 4B and D show non-endometrioid cancer compared with corresponding tumour-adjacent tissue compared with proliferative tissue. The proliferative tissue spectra used for comparison were the same in both. On histological examination, the tumour-adjacent tissue was benign and atrophic in the vast majority of cases (see Table 1).

Figure 4A shows clustering of all three groups and separation between endometrioid cancer and tumour-adjacent tissue. Here, the points (representing spectra) from proliferative tissue overlap with the other two categories, predominantly overlying the tumour-adjacent tissue plus a small area where all three categories are present. In contrast, Figure 4B shows less clear separation between the tumour and tumour-adjacent spectra. On close inspection, there appears to be two clusters within the non-endometrioid cancer category. The smaller of these groups is mingled with the tumour-adjacent and proliferative points and consists of spectra from two patients with uterine serous carcinoma. In such pathology, normal-looking cells under microscopy are likely to be at least partially tumourigenic; this approach in the future might even determine the tumourigenic potential of adjacent cell populations.

Figure 4C is the cluster vector plot derived from Figure 4A, comparing endometrioid cancer and tumour-adjacent tissue against proliferative endometrium. The most important wavenumbers in distinguishing between proliferative endometrium and endometrioid cancer are 1732 cm^−1^ (lipid), 1681/1620 cm^−1^ (both Amide I), 1581/1516 cm^−1^ (both Amide II), 1234 cm^−1^ (*ν*_as_PO_2_^−^), 1149 cm^−1^ (carbohydrate), 1020 cm^−1^ (glycogen), and 968 cm^−1^ (protein phosphorylation). The differences between proliferative endometrium and endometrioid tumour-adjacent tissue are much less pronounced.

In Figure 4D, the cluster vectors of non-endometrioid tumour-adjacent and cancer tissue are compared with normal proliferative endometrium. Unlike endometrioid cancer and its tumour-adjacent tissues, these cluster vectors are near-identical, representing similarity between non-endometrioid cancer and its corresponding adjacent tissue. The non-endometrioid cancer plot demonstrates a more pronounced single peak at 1736 cm^−1^ (lipid region) and a double peak at 1177 and 1146 cm^−1^ (?carbohydrate). The tumour-adjacent tissue has a larger peak at 1030 cm^−1^ (glycogen).

To strengthen the validity of our findings, all benign tissue (divided into premenopausal and postmenopausal tissue, all subtypes) was compared with all tumour-adjacent tissue as another category; such an examination would lend insight into the integrity of our tissue sampling and verify our post-spectroscopy histological analysis of the tissue sections. For instance, one would reasonably expect parameters such as myometrial invasion, blood vessel invasion, and cervical involvement to give rise to a marked number of outliers in the tumour-adjacent tissue spectral points in the scores plot. The scores plot in Figure 4E illustrates the large degree of overlap between the three groups in the absence of such outliers (particularly so in the tumour-adjacent tissue cluster), with PCA–LDA essentially unable to separate them. The premenopausal cluster exhibits the only degree of separation and a small number of outliers, which is something one might reasonably expect as a characteristic of this category. The cluster vector plot in Figure 4F shows only small differences between the tumour-adjacent and postmenopausal tissues. The most significant of these is within the lipid band with a smaller amplitude negative peak at 1735 cm^−1^ for tumour-adjacent tissue than for postmenopausal tissue, whose peak was slightly shifted to 1739 cm^−1^. As the majority of tumours studied were from postmenopausal women, this similarity suggests that the tumour-adjacent tissue is predominantly biochemically normal or benign.

### Classification of tumour subtypes

**Figure 5:**
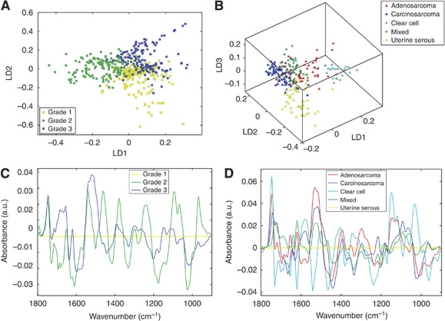
Comparison of grades of endometrioid carcinoma and subtypes of non-endometrioid carcinoma. (**A**) Endometrioid grades scores plot. (**B**) Non-endometrioid subtypes scores plot. (**C**) Endometrioid grades cluster vector plot (grade 1 as comparator). (**D**) Non-endometrioid subtypes cluster vector plot (uterine serous as comparator).

Tumours were subclassified according to their histological appearance. Endometrioid cancers were separated by grade and compared with one another (see Figure 5A and C), and the non-endometrioid cancers were compared separately (Figure 5B and D). Figure 5A shows separation between the three grades of endometrioid endometrial cancer. There are also regions of overlap between each grade and centrally between all three. Figure 5C compares the cluster vectors of grade 2 and grade 3 tumours against those of grade 1 tumours. Interestingly and surprisingly, grade 1 tumours appear more similar to grade 3 tumours than to grade 2. The most notable peaks in the cluster vector plot for grade 3 tumours are between 1800 and 1450 cm^−1^, that is, in the lipid region and in Amide I/II.

Figure 5B is a 3D scores plot, thus clustering and separation is dependent upon rotation. The clustered clear cell spectra separate clearly along LD1 from the amalgamated clusters of carcinosarcoma, uterine serous, and mixed spectra. The adenosarcoma spectra are dispersed between the two groupings. Along LD2, the uterine serous spectra separate from the carcinosarcoma, adenosarcoma, and clear cell spectra. LD3 separates the mixed group from all apart from adenosarcoma. When viewed in 3D, tight clusters are formed by the clear cell and carcinosarcoma groups. The uterine serous spectra are more spread out and overlap with the clear cell spectra. The mixed and adenosarcoma spectra, while still forming clusters, are more diffuse again and overlap with several other groups. Figure 5D compares uterine serous against all the other non-endometrioid tumour types. Adenosarcoma and carcinosarcoma appear alike in places; they have histological similarities as both contain glandular and connective tissue elements. Clear cell tumours are the most different from the other subtypes. Mixed tumours appear to have the most in common with uterine serous; as they often contain areas of uterine serous architecture, this is unsurprising.

## Discussion

This preliminary study demonstrates that ATR-FTIR spectroscopy has the potential to distinguish between benign and malignant endometrial tissues, and various subtypes thereof. It also appears capable of differentiating between normal tissues classified by phase of the menstrual cycle, although it was not able to distinguish between menstrual and proliferative tissues. This may be because the processing technique eliminates any loose surface shedding endometrium, revealing the very early (biochemically similar) proliferative tissue beneath. The most conspicuous differences are within the mid- and late-secretory phases, with a large accumulation of glycogen in the mid-secretory phase and rapid change in lipids in the late-secretory phase, together with structural protein changes indicated by several peaks in the Amide I/II areas. These changes are likely to be caused by preparation for the implantation of an embryo.

An important difference between tumour and benign tissue lies in the lipid area of the spectrum. The lipid absorbance in endometrioid cancers is greatest in grade 2 and 3 tumours, and among the non-endometrioid cancers in clear cell tumours, adenosarcomas and carcinosarcomas. This may represent qualitative or quantitative cell membrane changes. Further differences are present in the structural protein (Amide I/II) region. These are larger in endometrioid than non-endometrioid cancers, especially in Amide I. When tumours are compared with their corresponding tumour-adjacent tissue, a striking difference is observed between endometrioid and non-endometrioid cancers. In endometrioid cancer, the tumour-adjacent tissue almost resembles proliferative endometrium; in non-endometrioid cancer, it has more features in common with the adjacent cancer. This may be related to the pathogenesis of the two tumour types. Endometrioid cancer is strongly associated with unopposed oestrogen. This will produce proliferative changes in the non-cancerous adjacent tissue, perhaps too small to detect with histopathology, but discernable by spectroscopy.

Small areas of focal intraepithelial carcinoma in tumour-adjacent tissue could explain its similarity to the corresponding non-endometrioid tumour. However, Figure 4E suggests a good degree of clustering of spectral points derived from tumour-adjacent tissue pointing to this influence being minimal. Unfortunately, it was not possible to confirm this with conventional histopathology in the current study although all the tissue sections were H&E-stained and examined by a clinical pathologist post-spectroscopy. However, significant abnormality in the tumour-adjacent tissue is unlikely; when benign and tumour-adjacent tissues were compared, the categories were remarkably similar in the resultant scores plot (Figure 4E). Endometrioid carcinomas can be separated by grade, although there is a region of overlap on the scatter plot. Clear cell tumours are spectrally different from the rest of the non-endometrioid cancer group. They have a distinctive molecular signature, which they share with ovarian and renal clear cell disease (Zorn *et al*, 2005). It is not known whether they also share spectral similarities.

As malignant endometrial tissue differs fundamentally from normal tissue in terms of structure, genetics, and cellular activity, ATR-FTIR spectroscopy followed by multivariate analysis should be capable of distinguishing between them. Our data show some overlap between spectra from different tissue types, potentially leading to misclassification. The reasons for this are both methodological and biological. The method of ATR-FTIR spectroscopy followed by PCA–LDA described here was initially developed to distinguish between grades of cervical cytology (Walsh *et al*, 2007) and has since been applied successfully to prostate tissue (Martin *et al*, 2007). Principal component analysis–LDA is able to demonstrate data separation and highlight important wavenumbers; however, in its present form it is inadequate to predict the pathology of blindly presented samples. The total number of spectra obtained here (*n*=1999) is good for a preliminary study. When broken down to individual pathological entities, the numbers are small and the results, while promising, should be interpreted with caution. Alternative mathematical models and data processing methods are available and when combined with large training data sets more successful predictive models will be developed (Kelly *et al*, 2010).

The tissue collection and processing methodology was designed to be simple and reproducible. While it succeeds in this, a major drawback is that it is impossible to entirely exclude myometrial contamination. As myometrial spectra are likely to be similar regardless of the endometrial histological pattern, this could account for some of the areas of overlap. In future, this could be overcome by modifying the method of sample collection. Using either a suction catheter (e.g., Pipelle) or an endometrial brush (e.g., Tao brush) sampling device would significantly reduce the chance of harvesting myometrial tissue. It would also allow tissue collection from women not undergoing hysterectomy and to eliminate the need for a histopathologist at the point of sample collection. However, this method could not have been used to compare tumour with tumour-adjacent tissue as has been demonstrated here.

In a biological context, the fact that perfect segregation of cluster categories is not observed is unsurprising, as one would expect some degree of overlap associated with progression or relatedness of different cell types. This is, in part, because living material is seldom uniform and endometrial tissue is no exception. Tumour tissue, especially from a small or low-grade cancer, may contain a significant proportion of normal cells. In addition, the tissue examined by the histopathologist may have a different pathological appearance from the tumour-adjacent tissue used for spectroscopy. It has been postulated that some high-grade tumours develop from low-grade lesions by accumulating oncogenic traits (Llobet *et al*, 2009). Tumours may therefore contain a mixed cell population within the high-grade lesion as it transforms from a low-grade precursor. The overlapping regions on the scores plots could be explained by a combination of the above factors.

The current gold standard in determining tumour type and grade is expert interpretation by a consultant histopathologist. Because of the complex and subjective nature of the task, opinions occasionally differ. Biospectroscopy is an emerging discipline that applies spectroscopic techniques developed by physical chemists to biological material. Rather than using structural differences, tissue is analysed at a molecular level and yields objective results. Such techniques are therefore ideal for gathering data complementary to the traditional histology described above. The precise pathological findings are pivotal in planning the treatment of patients with endometrial cancer and an objective test could have a valuable supplementary diagnostic role. This technique may be particularly suitable as a screening technique for endometrial biopsies, giving a yes/no/maybe to the presence of cancer, thus determining the need for further investigation. This could prove useful in resource-poor settings where expert pathological services are limited.

## Figures and Tables

**Table 1:** Hierarchical classification system and subgroup numbers as spectra, samples, and patients

**Tissue classification**	**Spectra (*n*)**	**Samples (*n*)**	**Patients (*n*)**
*Normal*			
*Premenopausal*			
Proliferative	250	25	13
Early secretory	80	8	4
Mid secretory	20	2	1
Late secretory	60	6	3
Menstrual	61	6	3
On progesterone	50	5	3
*Postmenopausal*			
Atrophic	219	22	11
Other	40	4	2
			
*Cancer*			
*Endometrioid*			
Grade 1	179	18	9
Grade 2	140	14	7
Grade 3	140	14	7
*Non-endometrioid*			
Uterine serous	60	6	3
Clear cell	20	2	1
Mixed	40	4	2
Carcinosarcoma	80	8	4
Adenosarcoma	40	4	2
			
*Tumour adjacent*			
*Endometrioid (adjacent)*			
Atrophic	240	24	12
Hyperplasia	80	8	4
*Non-endometrioid (adjacent)*			
Atrophic	200	20	10
Hyperplasia	0	0	0

Abbreviation: *n*=number.

**Table 2:** Distinguishing wavenumbers (cm^−1^) of endometrioid and non-endometrioid cancers *vs* normal benign endometrium with likely originating bond-type

**Endometrioid cancer (cm^−1^)**	**Non-endometrioid cancer (cm^−1^)**	**Tentative assignment**
1736	1736	Lipid
1682		Amide I
1624	1624	Amide I
	1601	Amide I
1570	1570	Amide II
1516	1516	Amide II
1477	1477	?Protein
1450		?Protein
	1447	?Protein
1373		COO-symmetric stretching
1234		ν_as_PO_2_^−^
	1231	ν_as_PO_2_^−^
	1173	Carbohydrate
1169		Carbohydrate
	1142	C–O stretch (nu CO)
1088		ν_s_PO_2_^−^
1061	1061	ν_s_PO_2_^−^
1034		Glycogen
	1003	Glycogen
	968	Protein phosphorylation
964		Protein phosphorylation

?=possibly.
